# The Role of Cone Beam Computed Tomography (CBCT) in the Diagnosis and Clinical Management of Medication-Related Osteonecrosis of the Jaw (MRONJ)

**DOI:** 10.3390/diagnostics14161700

**Published:** 2024-08-06

**Authors:** Yui Yin Ko, Wei-Fa Yang, Yiu Yan Leung

**Affiliations:** Oral and Maxillofacial Surgery, Faculty of Dentistry, The University of Hong Kong, Hong Kong SAR, China; u3507133@connect.hku.hk (Y.Y.K.); teddyrun@hku.hk (W.-F.Y.)

**Keywords:** cone beam computed tomography, CBCT, medication-related osteonecrosis of the jaws, MRONJ, jaw necrosis, antiresorptive drug

## Abstract

Medication-related osteonecrosis of the jaw (MRONJ) is a debilitating condition associated with antiresorptive and antiangiogenic medications that are frequently used in treating osteoporosis and cancers. With the ability to produce high-resolution images with a lower radiation dose, cone beam computed tomography (CBCT) is an emerging technology in maxillofacial imaging that offers several advantages in evaluating MRONJ. This review aims to summarise the radiological features of MRONJ as observed via CBCT and highlight its advantages over two-dimensional plain films in assessing MRONJ. CBCT has the capability to detect early MRONJ lesions, characterise the extent and nature of lesions, distinguish MRONJ from other osseous pathologies, and assist in treatment planning. By leveraging the advantages of CBCT, clinicians can enhance their understanding of MRONJ, improve decision making, and ultimately optimize patient care.

## 1. Introduction

Medication-related osteonecrosis of the jaw (MRONJ) is a debilitating condition associated with antiresorptive and antiangiogenic medications that are frequently used in treating osteoporosis and cancers. MRONJ can be diagnosed based on the three criteria outlined in the position paper published by the American Association of Oral and Maxillofacial Surgeons (AAOMS): (1) current or previous treatment with antiresorptive therapy alone or in combination with immune modulators or antiangiogenic medications; (2) exposed bone or bone that probes through an intraoral or extraoral fistula in the maxillofacial region that has persisted for more than 8 weeks; and (3) no history of radiation therapy carried out on the jaws or no metastatic disease relative to the jaws [1].

The precise pathophysiology behind MRONJ remains inconclusive to date, but it has been hypothesised to be multifactorial in nature. Antiresorptive medication have been shown to exert inhibitory properties on bone turnover and angiogenesis, and they exert direct cytotoxic effects on osteoclasts. Along with the presence of active infection and inflammation, this produces a synergistic effect that accelerates the process of osteoclast apoptosis and subsequent necrosis. Dentoalveolar operations are the most common predisposing event for developing MRONJ. The risks of developing MRONJ after tooth extraction were found to range from 1.6% to 14.8% among oncological patients and from 0 to 0.15% among osteoporotic patients [1]. The risks of developing MRONJ following dental implants are still unclear. On the other hand, the failure rates of dental implants among patients at risk of MRONJ were found to be as high as 23%, with 83% of implant failures attributed to MRONJ development [2]. The presence of active infection is also increasingly recognized to be a crucial predisposing factor. Poor oral hygiene statuses can be frequently observed among those diagnosed with MRONJ [3], and chronic odontogenic infection induced by substandard root canal treatments has been known to trigger MRONJ development [4].

The condition of MRONJ has serious negative implications for patients’ quality of life. Not only does it exacerbate the burden of concomitant diseases, but patients also suffer from chronic pain and neurosensory disturbances, recurrent infections, and functional difficulties [5,6]. Psychologically, patients with MRONJ were more likely to experience symptoms of anxiety and depression [7]. The management of MRONJ aims to provide symptomatic control, eliminate infections, and limit necrosis progression. These range from conservative to surgical approaches that remove the necrotic bone. In advanced cases, radical resection and subsequent reconstruction might be required, which has been shown to significantly improve patients’ quality of life [8]. However, co-existing co-morbidities and advanced oncological status may preclude extensive surgical options, resulting in suboptimal outcomes.

Since the introduction of cone beam devices for the dento-maxillofacial region by Mozzo et al., the use of cone beam computed tomography (CBCT) has gained substantial popularity in the field of oral and maxillofacial surgery [9]. Compared to computed tomography (CT) imaging, CBCT can provide images with a better spatial resolution and high isotropic voxel resolutions ranging from 0.07 to 0.4 mm [10]. Another advantage of CBCT is its lower radiation dosage. Depending on the field of view and equipment protocol, the applied radiation dose is between 3% and 20% of a CT scan [11]. Other advantages of CBCT include easier accessibility with in-office imaging and reduced costs [12].

One of the major challenges posed by MRONJ is the difficulty in achieving early diagnosis and accurate staging. The early stages of MRONJ can present with symptoms resembling odontogenic infections or may even be asymptomatic. Furthermore, the clinical presentation often underestimates the true extent of the osseous disease. Therefore, high-quality adjunctive radiographic assessment is pivotal for all patients with suspected MRONJ in order to truly evaluate the extent of necrotic bone and determine the surgical intervention required. Additionally, the risks of MRONJ development in patients taking antiresorptive drugs (ADRs) remain undefined. Three-dimensional imaging techniques, such as CBCT, can enable the detection of early bony alterations in these patients. This can help identify radiographic features that may serve as risk predictors of MRONJ development. Hence, the role of CBCT can form a crucial element in the diagnostic algorithm for the management of MRONJ and the identification of high-risk patients.

In this article, we will explore the role of CBCT as an imaging modality for the diagnosis, prevention, and management of MRONJ.

## 2. Radiographic Features of MRONJ on CBCT

**Figure 1:**
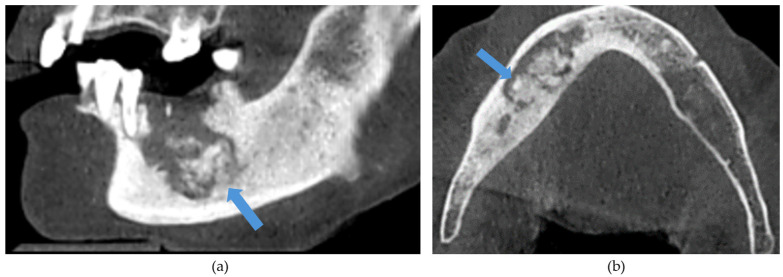
(**a**) Sagittal and (**b**) axial sections demonstrating an osteolytic lesion with sequestrations in the left mandible. Arrows demonstrate the sequestrations.

**Figure 2:**
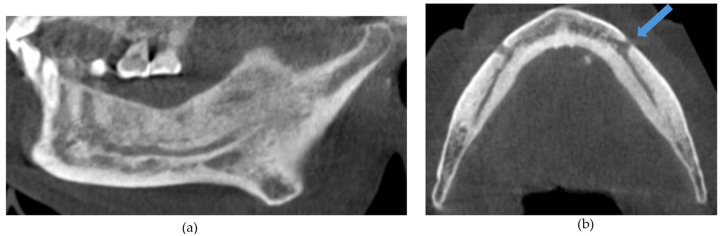
(**a**) Sagittal and (**b**) axial sections demonstrating generalized osteosclerosis of the mandible. Arrow demonstrates mild mental foramen narrowing.

**Figure 3:**
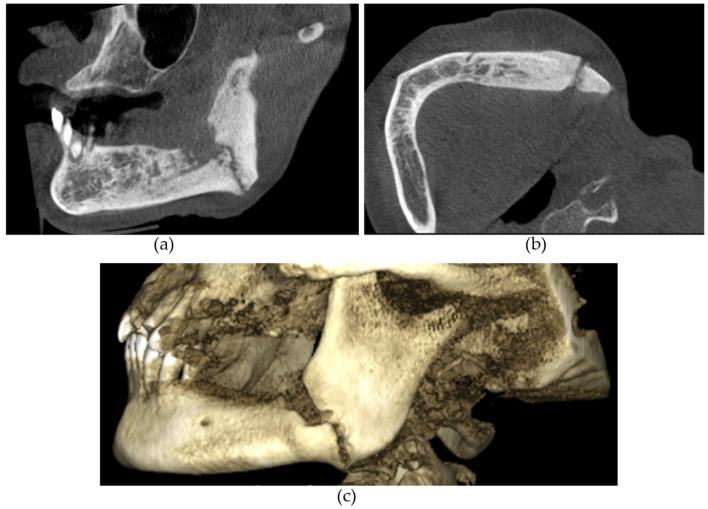
(**a**) Sagittal and (**b**) axial sections and (**c**) 3D reconstructed image demonstrating a left angle of the mandible pathological fracture resulting from stage III MRONJ.

**Figure 4:**
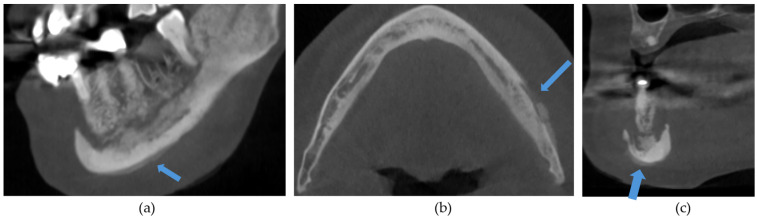
(**a**) Sagittal, (**b**) axial, and (**c**) coronal sections demonstrating a periosteal reaction (arrows) in the right posterior mandible.

**Figure 5:**
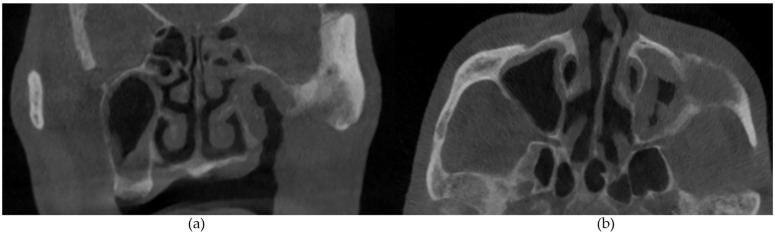
(**a**) Coronal and (**b**) axial sections demonstrating left maxillary sinus mucosal thickening. Extensive bony destruction of the maxillary alveolus, maxillary sinus walls, and zygoma can also be observed.

The CBCT findings commonly observed in patients with MRONJ are osteosclerosis, osteolysis, periosteal reaction, and sequestration [13,14,15,16,17,18]. The histomorphological analyses of MRONJ lesions have revealed three distinct patterns: (1) prominent bone resorption in regions of active inflammation, (2) acellular necrotic sequestra with large Haversian canals, and (3) increased inter-osteonic bone deposition with smaller Haversian canals and trabecular thicknesses in non-necrotic regions [19]. Abnormal bone remodellling characterised by reduced osteoclastic activity and increased appositional osteogenesis contributes to the radiological pattern observed in MRONJ lesions. In earlier stages of MRONJ, non-healing extraction sockets, the thickening of lamina dura, and periodontal ligament (PDL) space widening may also be observed [14,16,18,20]. In maxillary MRONJ, more prominent maxillary sinus mucosal thickening can be observed, although no significant differences in sinus volumes have been noted [21]. These radiological manifestations are illustrated in Figure 1, Figure 2, Figure 3, Figure 4 and Figure 5.

The availability of three-dimensional information on CBCT has improved our understanding of the evolution of MRONJ. Based on the radiological observations in patients on intravenous (IV) bisphosphonates, Barragan-Adjemian et al. have developed a model to illustrate the development from a non-exposed variant to a clinically exposed variant [22]. The model demonstrates the formation of involucrum(s), likely representing necrotic bone, inside the trabeculae of the sclerotic mandibular bone. An immune response leads to a resorptive circumference around the involucrum, which slowly increases with time. The involucrum then migrates following the path of least resistance, typically towards the edentulous area or lingually, resulting in exposed bone recognized clinically as sequestration.

MRONJ-associated osseous dimensional changes have been evaluated. The mandibular cortical bone has been found to be thicker in MRONJ patients, particularly in the buccal and apical cortex [23,24]. The overall mandibular cortical bone volume, area, and ratio of mandibular cortical bone area to height are found to be elevated in MRONJ cases [25]. However, it should be noted that the mandibular cortical cortex is significantly thicker in both oncological and osteoporotic patients exposed to bisphosphonates. This suggests that this radiological sign may be induced by a reduction in the bone remodelling rate from bisphosphonate use rather than a radiological sign specific to MRONJ [24,26].

Mental neuropathy can manifest as a prodromal sign of MRONJ [27]. CBCT has been used to evaluate changes in the dimensions of neurovascular bundles. The narrowing of the mandibular foramen, mental foramen, lingual foramen, and incisive canal has been observed in regions affected by MRONJ [23,28]. However, no studies have specifically examined the presence of these radiological manifestations in relation to the presence of neurosensory disturbances and clinical staging.

## 3. Diagnosing MRONJ by Imaging

### 3.1. CBCT vs. Conventional Plain Radiographs in Radiological Assessment

Clinical assessment and conventional plain radiographs are routinely undertaken for all patients with suspected MRONJ. Radiographic evaluation is a crucial element in the diagnostic algorithm, enabling clinicians to determine the extent of bony changes; the size and location of sequestration; proximity to neurovascular bundles and maxillary sinuses; and the presence of a pathological fracture. It may also alert clinicians to radiological signs of malignancy relapse among oncological patients [29]. Although panoramic radiographs (PRs) provide an excellent overview of the jaws, mineral loss in osteolytic lesions must be as high as 30–50% to be clearly visible. Moreover, their two-dimensional nature limits their ability to accurately delineate the lesion and differentiate necrotic bone from healthy bone accurately [30].

CBCT has been found to be superior to PR in assessing the extent of lesions, particularly in advanced cases. A comparative study conducted on 22 MRONJ patients receiving IV bisphosphonates revealed that PR did not show apparent radiological changes in asymptomatic cases or those with denuded bone in the posterior lingual region. In contrast, CBCT was able to detect non-specific osteosclerotic changes in these cases. In advanced MRONJ cases, CBCT provided more detailed information about the cortical and cancellous bone involvement, proximity to neurovascular structures, depth and size of sequestra, and the presence of pathological fractures [20]. Studies by Demir et al. and Galiti et al. also highlighted that CBCT was more sensitive than PR in interpreting cortex irregularity, osteolytic changes, osteosclerosis, periosteal reaction, and sequestration across all stages of MRONJ, especially in the posterior mandible [18,31].

The need for CBCT evaluation is further emphasized in a case series of patients with pre-existing MRONJ prior to dental extractions [32]. In this series, 10 patients presented clinically with pain, swelling, and tooth mobility but no bone exposure. Plain film radiographs indicated features consistent with odontogenic infection, including periapical radiolucency, PDL space widening, and alveolar bone loss. The diagnosis of pre-existing MRONJ was only revealed when CBCT demonstrated necrotic patterns of bony destruction and sequestration.

### 3.2. Radiographic Findings on CBCT and Clinical Staging

Patients with MRONJ exhibit a range of disease severities, and accurate staging is crucial for determining appropriate treatment modalities. According to the AAOMS position paper, the clinically exposed bone variant can be categorised into three stages based on the presence of inflammation or infection, patients’ symptoms, and involvement with adjacent structures [1]. Furthermore, the clinical presentation of MRONJ may underestimate the extent of the disease compared to the radiological findings, highlighting the importance of radiological evaluation in treatment planning [33].

To investigate the correlation between radiological presentations and clinical staging, Wilde et al. conducted a retrospective study involving 27 patients [17]. The most common radiological presentations were the destruction of cortical and cancellous bone, and their occurrences appeared to increase with the severity of MRONJ. Sequestration and osteosclerosis were observed across all stages, while new periosteal bone formation was only present in advanced stages.

To objectively evaluate the extent and severity of MRONJ-related radiographic changes, a composite radiographic index was developed by Walton et al. [15]. The index is scored based on four radiographic parameters: sclerosis, lytic changes, periosteal reaction, and sequestration. The extent of radiographic changes is visually scored as follows: 0 for absence, 1 for changes localised within one tooth dimension mesiodistally from the site of clinical bone exposure and limited to the alveolus, and 2 for changes beyond these boundaries. The total score can range from 0 to 8 and can be classified as low (0–2), medium (3–5), or high (6–8). The index was further modified by Badabaan et al. by assigning a score of ‘3′ for extensive or diffuse radiographic changes to give more weightage to widespread radiographic changes, thereby enhancing its accuracy in association with clinical staging [34]. Low scores are mostly associated with stage I cases, while high scores are seen in stage 3 cases [15].

Accurate radiographic staging, as facilitated by the composite radiographic index, provides valuable insights into the severity of MRONJ and guidance in selecting appropriate treatment modalities.

### 3.3. Differentiation between Different Osseous Pathological Conditions

The radiological manifestations of MRONJ exhibit similarities with other osseous pathological entities, such as osteoradionecrosis, osteomyelitis, and metastasis to the jaw. These conditions can often appear to be indistinguishable on two-dimensional PRs. However, the utilisation of three-dimensional imaging provided by CBCT enables the identification of subtle differences that aid in their differentiation.

CBCT imaging has revealed that larger sclerotic areas and periosteal bone formation are more frequently observed in MRONJ lesions compared to osteoradionecrosis and osteomyelitis [35,36]. Non-healing extraction sockets and sequestration are also more common in MRONJ compared to osteomyelitis and jaw metastasis [36]. Lytic lesions and pathological fractures are more prevalent in MRONJ compared to osteomyelitis [35]. In contrast, PRs can only highlight the differences in cortical bone resorption, PDL space widening, and the thickness of the lamina dura between MRONJ, osteomyelitis, and osteoradionecrosis [35]. Consequently, CBCT has a higher predictive value of 90% in differentiating these three entities compared to PR, which has a predictive value of 74% [35]. The frequency of the occurrence of radiological features in different osseous pathologies is summarised in Table 1.

### 3.4. Radiological Differences between Different Underlying Medical Conditions and Medications

The two classes of antiresorptive medications implicated in MRONJ are bisphosphonates and denosumab; both act on a cellular level to inhibit osteoclastic activity. However, the mechanisms of action and half-lives of these medications were vastly distinct. Bisphosphonates have a high affinity for hydroxyapatite (HAP) in bones, allowing them to closely interact with mature osteoclasts and exert its inhibitory properties [37,38]. Nitrogen-containing bisphosphonates inhibit the production of enzymes essential for osteoclast function and survival [37,39], while non-nitrogen-containing subtypes are metabolized by osteoclasts to induce apoptosis [37,38]. On the other hand, denosumab is a human monoclonal antibody that binds with high affinity to RANKL, inhibiting the activation of RANK receptors on the surfaces of osteoclast precursors and osteoclasts. This thereby inhibits osteoclast formation and function [40]. Due to their differences in mechanisms, denosumab has a half-life of 26 days, whereas the half-lives of bisphosphonates can range from 1 to 10 years [40,41].

Consequently, it has been speculated that the radiological characteristics of MRONJ lesions could differ depending on the type of medication administered. In a retrospective study of 34 patients with MRONJ, Pichardo et al. found that sequestration and the lysis of the cortical border were found to be 30% less prevalent among patients on denosumab compared to those on bisphosphonates [13]. Additionally, non-exposed variant sites exposed to bisphosphonates demonstrated more osteosclerosis than those exposed to denosumab [42]. Hence, it has been hypothesized that this difference may lead to the underdiagnosis and undertreatment of denosumab-related osteonecrosis of the jaw.

On the other hand, evidence regarding the radiological differences between different underlying diseases remains inconclusive. Muttanahally et al. evaluated the CBCT of 12 oncological patients on antiresorptive medication [14]. However, due to limitations on sample size, no distinctive radiological features could be observed in relation to the patient’s primary cancer and the medication used. Walton et al. compared the radiographic characteristics of MRONJ between 41 oncological and 29 osteoporotic patients, yet no significant differences could be noted in terms of sclerosis, lytic changes, sequestration, and periosteal reaction [15].

In oncological patients with bone metastases, bisphosphonates were administered intravenously at a higher dosage and shorter interval compared to osteoporotic patients. Therefore, the route and duration of medication may affect the radiological presentation. Via the semiautomatic segmentation of CBCT images, Lentzen et al. and Zhou et al. obtained the volumetric measurement of osteolytic MRONJ lesions [43,44]. They found that patients with IV administration of bisphosphonates presented with significantly larger lesion volumes compared to those with oral and subcutaneous administration of denosumab. A retrospective review carried out by Pichardo et al. revealed that the severity of osteosclerosis depends on the duration and potency of bisphosphonate therapy [45]. This concurs with a study by Zhou et al., which demonstrated higher radiodensity values in osteosclerotic regions in patients with a longer duration of bisphosphonate administration [44].

## 4. Treatment Planning of MRONJ with CBCT

Identification of patients at risk of developing MRONJ and stage 0 lesions with CBCT.

Tooth extraction is a known triggering factor for the development of MRONJ, creating a therapeutic dilemma for clinicians. Identifying individuals at a higher risk of developing MRONJ can assist in the shared decision-making process between clinicians and patients, and it can enable the incorporation of adjunctive techniques during extraction, such as alveolectomy and the use of biological membranes to limit progression [46].

The AAOMS position paper describes a stage 0 non-exposed bone variant of MRONJ [1]. Stage 0 lesions may present with various non-specific symptoms, including jaw ache, sinus tract, bone enlargement, and gingival swelling. Approximately 50% of stage 0 MRONJ lesions progress to clinical bone exposure within 4.6 months [47]. However, the identification of these patients can be challenging. In the absence of mucosal breakdown, diagnosis relies on exclusion, making early diagnosis based solely on clinical examination difficult. Early recognition can facilitate the prompt removal of local provoking factors to limit disease progression and enable more vigilant monitoring.

CBCT offers clear advantages over two-dimensional plain films in evaluating osseous radiological changes in early MRONJ lesions and has demonstrated greater diagnostic sensitivity [48]. In a cohort of 130 patients with 237 treated extraction sites, CBCT displayed a superior detection rate of 87.4% for early non-vital bone changes compared to 26.8% in PRs [49].

CBCT has been shown to be a feasible predictive tool for detecting patients who are at risk of developing MRONJ following dental extractions. Catalina et al. conducted a longitudinal, case–control study comparing the pre-operative CBCT images of 47 oncologic patients on antiresorptive medications [42]. They found that the presence of localized and extensive periosteal reactions was a radiographic sign that predisposes patients to MRONJ. Moreover, as the presence of sequestra was found exclusively among patients on antiresorptive medications, they suggested that this may represent an underlying masked necrotic process and advocated the removal of these sequestra at the time of extraction to limit progression. This is further supported by a study carried out by Soundia et al. that included 20 stage 0 MRONJ patients, where 80% of the patients who did not progress to bone exposure did not present with sequestra [50]. This finding indicated that the absence of sequestra can be a reliable radiological predictor for conservative management.

### CBCT Assists in the Management of MRONJ

CBCT has been recognized for its role in influencing the management of MRONJ lesions. A questionnaire-based study by Kammerer et al. revealed that CBCT improved diagnostic sensitivity among maxillofacial surgeons in detecting osteosclerosis, bone remodelling, cortical bone continuity, and sequestration compared to PRs [11]. Subsequently, over 50% of the surgeons adjusted their surgical approach and assessed the need for an inpatient setting or general anaesthesia based on the CBCT findings.

Given the unclear pathophysiology and systemic complexities of patients with MRONJ, the optimal treatment, whether conservative versus surgical, remains debatable. Thus, CBCT images could provide radiographic prognostic factors in guiding treatment decisions. Rabie et al. analysed the radiographic tomographic appearance of 143 MRONJ lesions from 115 patients treated with antiresorptive medications and examined their subsequent outcomes [51]. Among patients treated conservatively, extensive osteosclerosis, deep sequestra formation, and tooth involvement were found to be poor prognostic factors. Therefore, the authors suggested that teeth removal and sequestrectomy may be advocated to enhance mucosal healing. On the other hand, among patients treated with sequestrectomy and antimicrobial therapy, the absence of sequestrum formation and the presence of periosteal reaction were found to be poor prognostic indicators for mucosa healing. Hence, the authors proposed that these radiographic signs may prompt the need for a more extensive resection.

Another challenge encountered during the treatment of MRONJ is accurately determining the extent of the lesion. The extent of surgical debridement and block resection is determined intra-operatively based on the presence of bleeding viable bones and bone morphology. Undertreatment poses a higher risk of treatment failure and recurrence, while overtreatment is associated with increased morbidity and the need for advanced reconstruction. Subramanian et al. hypothesised the use of a digital fusion of functional imaging and CBCT to demarcate MRONJ lesions. The integration of fluorodeoxyglucose positron emission tomography/diffuse single-photon emission computed tomography (FDG-PET/SPECT) and CBCT allows the evaluation of bone remodelling and inflammation activity, as well as the provision of anatomic precision for surgical planning [52]. This technique has been applied in a case where a patient remained asymptomatic 8 months following limited marginal mandibulectomy, demonstrating its success.

## 5. Follow-Up and Monitoring of Progression with CBCT

Following clinical interventions, the use of CBCT could be valuable in monitoring disease progression. Radiological parameters have been developed to quantitatively assess the changes in bone quality based on the alteration in X-ray attenuation. Radiodensity and volumetric measurements of MRONJ lesions provide a more objective quantitative comparison and clearer visualisation of disease progression [44,53].

Fractal dimension (FD), which quantifies the complexity and irregularity of surfaces, has been applied to analyse structural changes within the trabecular bone. In the MRONJ-affected maxilla and mandible, the FD value is lower compared to healthy individuals due to the development of osteosclerosis in cancellous bone [54]. It has been hypothesized that FD analysis could serve as a potential tool for the early detection of bony changes in MRONJ lesions.

Measurements of bone mineral density (BMD) can also be achieved by quantifying the sizes and grey value of image pixels on CBCT [55]. Affected areas with MRONJ have been shown to demonstrate higher BMD values compared to ipsilateral and contralateral non-affected areas, with the highest value noted adjacent to MRONJ lesions [56]. Intracortical bone density values were also observed to be lower in MRONJ patients compared to healthy individuals [23]. In a study by Zhou et al., the radiodensity values of post-operative CBCT imaging were compared in 41 patients [44]. It was found that radiodensity values of post-surgical lesions with recurrence were higher than those without recurrence. This finding may allow the identification of patients with a higher risk of recurrence, enabling clinicians to implement more vigilant clinical and radiological follow-ups.

Advances in the understanding of wound healing growth factors hold promise in promoting bony regeneration in the management of MRONJ. CBCT can be utilised to provide a three-dimensional assessment when evaluating the efficiency of these treatments. Pardinas-Lopez et al. reported three cases in which MRONJ was treated with surgical debridement of the necrotic bone and regeneration with platelet-rich growth factor (PRGF) [57]. Subsequent surveillance CBCT demonstrated a bone volume gain of 12% to 30% twelve months post-operatively.

Post-operative bony regeneration using recombinant human bone morphogenetic protein-2 (rhBMP-2) has also been reported to be successful [58,59]. In a study by Jung et al., the local application of rhBMP-2 and short-term adjunct teriparatide administration enhanced bone formation in MRONJ-associated bony defects significantly compared to only the local administration of rhBMP-2 and control groups 6 months post-operatively [59]. CBCT findings revealed that the bone regeneration ratio was over 50% higher in the treatment group compared to the control group. Additionally, this study observed that deeper and narrower defects exhibited a more rapid rate of bone regeneration compared to flat and shallow defects. These findings, derived from CBCT imaging, can help facilitate personalized therapy approaches and improve case selection, ultimately optimizing success rates in the management of MRONJ.

In short, CBCT plays a crucial role in monitoring disease progression, assessing treatment outcomes, and optimizing therapeutic approaches in the management of MRONJ.

## 6. Comparison with Other Three-Dimensional Imaging Modalities (Table 2)

While CBCT is an asset in the assessment of MRONJ, it does have limitations when it comes to soft tissue evaluation. Its ability to discriminate soft tissue is limited compared to magnetic resonance imaging (MRI) and contrast CT scans, which may result in an underestimation of soft tissue involvement and inflammation [60]. Another limitation of CBCT is its inability to detect bone remodelling during the early stages of disease manifestation. Its ability to detect inflammation and bone metabolism is limited compared to PET/CT scans; hence, PET/CT scans may be superior to CBCT in detecting subclinical MRONJ lesions [61].

When comparing the utility and efficiency of CBCT with other three-dimensional imaging modalities, a study involving 19 patients found that CBCT and ultrashort echo-time MRI were comparable in detecting osteolysis, periosteal thickening, and osteosclerosis [62]. However, MRI was prone to image distortion by motion artifacts and beam hardening caused by dental hardware. Furthermore, air-induced artifacts on ultrashort echo-time MRI could be mistaken for periosteal bone reaction or sequestration.

Another study compared the effectiveness of CBCT, contrast-enhanced MRI, and [18F] fluoride positron emission tomography–computed tomography (PET/CT) in evaluating the extent of MRONJ lesions by comparing the findings with intraoperative observations in 10 patients [63]. While all three imaging modalities were more accurate than clinical examination alone, contrast-enhanced MRI and [18F] fluoride PET/CT tended to overestimate lesion sizes, while CBCT tended to underestimate the extent of the lesion.

**Table 2:** Comparison of different imaging modalities in the assessment of MRONJ lesions as deduced by the data presented by Arce et al. [64].

Imaging Modality	Radiation Dosage	Cost	Scanning Time	Role in Assessment
Plain films	Low	Low	<1 min	Initial screening
CBCT	Medium	Medium	<1 min	Detecting trabecular bone changes, presence of bone erosion and sclerosis, sequestration, and periosteal bony reaction
CT	High	Medium	3–10 min	
MRI	N/A	High	20–40 min	Detecting involvement of the bone marrow, surrounding soft tissues, neurovascular bundles, lymphadenopathy
PET-CT	Very high	Very high	60–90 min	Differentiation between necrotic regions and viable bone

PET: Positron Emission Tomography.

These findings highlighted the importance of considering the strengths and limitations of different imaging modalities when evaluating MRONJ. The choice of imaging should be based on the specific clinical scenario and the information required for accurate diagnosis and treatment planning.

## 7. Limitations and Future Directions

Overall, there are a few limitations in the current literature surrounding MRONJ. Firstly, due to the low incidence rate of MRONJ, most studies are of a retrospective observational nature with limited sample sizes. Secondly, clinical heterogeneity in a study group is often observed with regard to the patient’s underlying comorbidities, antiresorptive medication type and dosage, surgical approaches, and use of adjuncts. Thirdly, parameters for evaluating treatment outcomes remain poorly defined and inconsistent across the literature. The need for well-designed randomised controlled clinical trials is required to establish evidence-based treatment algorithms.

A lack of consensus over prevention and management remains a major topic of debate; future clinical studies could utilise CBCT to provide insights into such prospects. As the presence of sequestration in pre-extraction CBCT was identified as a risk factor for MRONJ occurrence, further studies are necessitated to ascertain the value of pre-extraction CBCT in MRONJ prevention. Risk stratification models based on radiological presentation may be developed to identify patients at higher risks of developing MRONJ following dentoalveolar surgeries.

The determination of resection margins in MRONJ lesions remains controversial. Current AAOMS guidelines recommend the boundaries of radical resection to be based on the radiological involvement of the inferior alveolar canal and maxillary sinus. However, MRONJ lesions frequently present with widespread sclerotic bone appearance. If this were to be included as part of the resection, it may result in overtreatment. A recent study by Obermeier et al. observed no significant differences in the radiodensity of pre-operative and post-operative panoramic sclerotic regions in MRONJ lesions; hence, they hypothesised that sclerotic bones are reactive in nature and should not be routinely removed [65]. Future studies comparing radiodensity values with three-dimensional pre-operative and post-operative imaging may be beneficial in delineating bony resection margins. This may also facilitate the integration of virtual surgical planning.

Lastly, the evaluation of bone remodelling following the use of regenerative therapies and growth factors in treating MRONJ defects is limited to case series. A large-scale study quantifying bony defects and subsequent bony regeneration using CBCT could potentially allow a better evaluation of treatment efficacy.

## 8. Conclusions

CBCT is an emerging technology in the field of maxillofacial imaging and is undoubtedly a valuable imaging modality in the assessment and management of MRONJ. Its three-dimensional visualisation capabilities provide significant advantages over two-dimensional plain films, allowing for a comprehensive evaluation of MRONJ lesions, especially in advanced cases. The high spatial resolution of CBCT images enables the identification of early bony trabecular changes, which can be crucial in identifying individuals at risk or at early stages of MRONJ. Additionally, CBCT allows for the characterisation of MRONJ lesions, aiding in the differentiation of MRONJ from other osseous pathologies, influencing treatment planning, and providing prognostic markers for clinicians. Overall, CBCT plays a vital role in the understanding, diagnosis, and management of MRONJ.

## Figures and Tables

**Table 1:** Frequency of occurrence of radiological features in osseous pathologies as deduced by the data presented by Gaeta-Araujo et al. [35] and Yfanti et al. [36].

Radiological Sign	MRONJ	Osteoradionecrosis	Osteomyelitis
Osteosclerosis	++	+	++
Osteolysis	++	++	+/−
Periodontal ligament space widening	+	+/−	+
Lamina dura thickening	+/−	+/−	+
Sequestration	++	++	+
Pathological fracture	+	+	+/−
Periosteal reaction	+	+/−	++

(++): Typically present; (+): may be present, (+/−): occasionally present.

## Data Availability

No new data were created or analysed in this study. Data sharing is not applicable to this article.
